# Comparison of matched sibling donors versus unrelated donors in allogeneic stem cell transplantation for primary refractory acute myeloid leukemia: a study on behalf of the Acute Leukemia Working Party of the EBMT

**DOI:** 10.1186/s13045-017-0498-8

**Published:** 2017-06-24

**Authors:** Eolia Brissot, Myriam Labopin, Matthias Stelljes, Gerhard Ehninger, Rainer Schwerdtfeger, Jürgen Finke, Hans-Jochem Kolb, Arnold Ganser, Kerstin Schäfer-Eckart, Axel R. Zander, Donald Bunjes, Stephan Mielke, Wolfgang A. Bethge, Noël Milpied, Peter Kalhs, Igor-Woflgang Blau, Nicolaus Kröger, Antonin Vitek, Martin Gramatzki, Ernst Holler, Christoph Schmid, Jordi Esteve, Mohamad Mohty, Arnon Nagler

**Affiliations:** 1 Service d’Hématologie Clinique et de Thérapie Cellulaire, Hôpital Saint Antoine, APHP, 184 rue du faubourg Saint-Antoine, 75571 Paris, Cedex 12 France; 2 Acute Leukemia Working Party Office, Hôpital Saint Antoine, APHP, Paris, France; 3 Department of Medicine A/Hematology and Oncology, University of Muenster, Muenster, Germany; 4 Medizinische Klinik und Poliklinik I, Universitätsklinikum, Dresden, Germany; 5 Deutsche Klinik für Diagnostik, KMY, Zentrum, Wiesbaden, Germany; 6 Faculty of Medicine and Medical Center, Hematology, Oncology and Stem Cell Transplantation, University of Freiburg, Freiburg im Breisgau, Germany; 7 Klinikum Grosshadern, Med. Klinik III, München, Germany; 8 Department of Hematology, Hemostasis, Oncology and Stem Cell Transplantation, Hannover Medical School, Hannover, Germany; 9 Medizische Klinik, Paracelsus Medizinische Privatuniversität, Nürnberg, Germany; 10 Bone Marrow Transplantation Center, University Hospital Eppendorf, Hamburg, Germany; 11 Klinik fuer Innere Medizin III, Universtätklinikum, Ulm, Germany; 12 Department of Internal Medicine II, Würzburg University Medical Center, Würzburg, Germany; 13 Medical Department, Hematology and Oncology, University of Tuebingen, Tübingen, Germany; 14 Hematology, CHU de Bordeaux, Bordeaux, France; 15 Department of Internal Medicine I, Bone Marrow Transplantation Unit, Medical University of Vienna, Vienna, Austria; 16 Charite-Campus Benjamin Franklin Universitaetsmedizin Berlin Klinik III- Hematologie u Onkologie, Hindenburgdamm, Berlin, Germany; 17 Department of Stem Cell Transplantation, University Medical Center Hamburg-Eppendorf, Hamburg, Germany; 18 Department of Clinical Hematology, Institute of Hematology and Blood Transfusion, Prague, Czech Republic; 19 Division of Stem Cell Transplantation and Immunotherapy, University of Kiel, Kiel, Germany; 20 Department of Haematology/Oncology, University Hospital Regensburg, Regensburg, Germany; 21 Medizinische Klinik Klinikum, Augsburg, Germany; 22 Hospital Clinic Institut d’investigacions Biomèdiques August Pi i Sunyer, Barcelona, Spain; 23 Chaim Sheba Medical Center, Tel Hashomer, Israel

**Keywords:** Acute myeloid leukemia, Refractory, Allogeneic stem cell transplantation, HLA-matched related donor, Unrelated donor, Graft-versus-host disease

## Abstract

**Background:**

Primary refractory acute myeloid leukemia (PRF-AML) is associated with a dismal prognosis. Allogeneic stem cell transplantation (HSCT) in active disease is an alternative therapeutic strategy. The increased availability of unrelated donors together with the significant reduction in transplant-related mortality in recent years have opened the possibility for transplantation to a larger number of patients with PRF-AML. Moreover, transplant from unrelated donors may be associated with stronger graft-mediated anti-leukemic effect in comparison to transplantations from HLA-matched sibling donor, which may be of importance in the setting of PRF-AML.

**Methods:**

The current study aimed to address the issue of HSCT for PRF-AML and to compare the outcomes of HSCT from matched sibling donors (*n* = 660) versus unrelated donors (*n* = 381), for patients with PRF-AML between 2000 and 2013. The Kaplan-Meier estimator, the cumulative incidence function, and Cox proportional hazards regression models were used where appropriate.

**Results:**

HSCT provide patients with PRF-AML a 2-year leukemia-free survival and overall survival of about 25 and 30%, respectively. In multivariate analysis, two predictive factors, cytogenetics and time from diagnosis to transplant, were associated with lower leukemia-free survival, whereas Karnofsky performance status at transplant ≥90% was associated with better leukemia-free survival (LFS). Concerning relapse incidence, cytogenetics and time from diagnosis to transplant were associated with increased relapse. Reduced intensity conditioning regimen was the only factor associated with lower non-relapse mortality.

**Conclusions:**

HSCT was able to rescue about one quarter of the patients with PRF-AML. The donor type did not have any impact on PRF patients’ outcomes. In contrast, time to transplant was a major prognostic factor for LFS. For patients with PRF-AML who do not have a matched sibling donor, HSCT from an unrelated donor is a suitable option, and therefore, initiation of an early search for allocating a suitable donor is indicated.

**Electronic supplementary material:**

The online version of this article (doi:10.1186/s13045-017-0498-8) contains supplementary material, which is available to authorized users.

## Background

Primary refractory acute myeloid leukemia (PRF-AML) remains a major therapeutic challenge. There is no agreed definition of PRF-AML, it has been defined by the absence of complete remission (CR), manifested by blast count of ≥5% in bone marrow after one or two cycles of intense induction therapy [1–3]. Recently, less than a 50% reduction in blast numbers with >15% residual blasts after one cycle of induction chemotherapy permits the early identification of patients whose outcome is very poor [4]. CR is achieved in 10–40% of the cases depending on the AML patient population. Allogeneic stem cell transplantation (HSCT) is the only salvage option with true curative potential in this scenario [5–8]. In the past decade, the use of reduced intensity conditioning regimen (RIC) has significantly increased the proportion of patients with PRF-AML who are eligible for transplant. Indeed, it has been demonstrated that HSCT from matched sibling donors (MSD) is a valid option, leading to disease-free survival rates that are in the range of 20–30% in these very high risk devastated patient population [7, 9–11]. More recently, PRF-AML were offered HSCT from unrelated donors for (UD), with an overall survival rate (OS) in the range of 22% [12, 13]. Moreover, HLA mismatch and minor histocompatibility antigen mismatch, which is assumed to be present in fully matched unrelated and also in fully matched related donors, augment donor/recipient alloreactivity, and it has been postulated that histo-incompatibility between unrelated donors and their recipients may be associated with augmented graft-versus-leukemia effect (GVL) and hence lower risks of relapse [14]. However, the various studies comparing the outcomes of AML patients with MSD or UD yielded controversial results, some reporting inferior survival or disease-free survival with UD HSCT [15–18], while others concluded to similar survival rates [19, 20]. One of the largest AML studies reported similar survival times with MSD and matched UD [21]. Notably, only small series are available concerning PRF-AML. Advances in RIC have significantly increased the proportion of patients with refractory AML who are eligible for transplant. Craddock et al. reported 36 patients who received transplant with a RIC with an UD with an OS at 36% at 5 years similar to 18 patients with myeloablative conditioning regimen (MAC) [12]. In the past decade, it has been demonstrated that improvement of outcomes where related to the reduction of non-relapse mortality (NRM), majority due to lower infections, lower organ damages, and reduction of severe acute graft-versus-host disease (aGVHD) [22]. Because the success of UD HSCT is significantly influenced by the degree of high-resolution HLA matching [23] and because advances in supportive care influence outcomes [22, 24, 25], a safety and efficacy comparison of UD versus MSD HSCT in a recent and large cohort of patients is highly warranted to further document decision-making. Therefore, present study based on the European Society of Blood and Marrow Transplantation (EBMT)—Acute Leukemia Working Party (ALWP) database in order to compare outcomes after MSD HSCT versus 10/10 or 9/10 HLA-matched UD HSCT for PRF-AML.

## Methods

### Study design and data retrieval

This is a retrospective, multicenter, registry-based analysis. Data for this study were provided and approved by the Acute Leukemia Working Party of the EBMT group registry. The EBMT registry is a voluntary working group of more than 600 transplant centers, mostly located in Europe, that are required to report all consecutive stem-cell transplantations and follow-up data once a year. Data are entered, managed, and maintained in a central database with internet access; each EBMT center is represented in this database. There are no restrictions on centers for reporting data, except for those required by the law on patient consent, data confidentiality, and accuracy. Quality control measures included several independent systems: confirmation of validity of the entered data by the reporting team, selective comparison of the survey data with MED-A data sets in the EBMT registry database, cross-checking with the National Registries, and regular in-house and external data audits. Since 1990, patients provide informed consent authorizing the use of their personal information for research purposes.

Eligibility criteria for this analysis included adult patients (aged >18 years) with PRF-AML in active disease who had received a first HSCT from HLA-identical sibling or an unrelated donor (9/10 or 10/10) with bone marrow or granulocyte colony-stimulating factor-mobilized peripheral blood stem cells. PRF-AML was defined by the failure of achieving CR (bone marrow blasts ≤5%) despite induction chemotherapy. All unrelated donors were HLA-matched (10/10) or mismatched at one loci (9/10) (-A, -B, -C, -DRB1, -DQB1). We excluded patients who had undergone haploidentical or umbilical cord blood HSCT so that the analysis was restricted to a more homogeneous study population. MAC was defined as per EBMT definition a regimen containing total body irradiation with a dose >6 Gy, a total dose of oral busulfan (BU) >8 mg/kg, or a total dose of intravenous BU >6.4 mg/kg [26]. The sequential strategy consisted of pre-transplant chemotherapy followed by a conditioning regimen [27]. All patients provided informed consent for transplants according to the declaration of Helsinki [26].

### End points

OS was calculated from the date of transplant until death or last observation alive. Leukemia-free survival (LFS) was calculated from the date of transplant until relapse or last disease-free follow-up. Relapse and death from any cause were considered events. NRM was defined as death without prior relapse. Neutrophil recovery was defined as achieving absolute neutrophil count greater than—or equal to—0.5 × 109/l for three consecutive days. The diagnosis and grading of acute [28] and chronic graft-versus-host disease [29] were performed by transplant centers using the standard criteria. Cytogenetics abnormalities were classified according to the European Leukemia Net cytogenetic classification system [30].

### Statistical analysis

Patient-, disease-, and transplant-related variables were compared between the two groups (MSD or UD) using the chi-square statistic for categorical variables and the Mann-Whitney test for continuous variables. Variables considered were patient’s age at transplantation, donor/recipient sex, interval from diagnosis to transplantation, cytogenetics group, type of conditioning (RIC/MAC/FLAMSA), source of stem cells (peripheral blood stem cell (PBSC) versus bone marrow (BM)), patient/donor CMV serology, Karnofsky performance status (KPS) at time of transplantation, in vivo T cell depletion, and year of transplantation. Factors that differ significantly between the two groups with *p* values of <0.05 and all factors known as potential prognostic factors were included in the final models. Cumulative incidence functions (CIF) were used to estimate RI and NRM in a competing risk setting, because death and relapse compete with each other. To study chronic graft-versus-host disease (cGVHD), we considered relapse and death to be competing events. Probabilities of LFS and OS were calculated using the Kaplan-Meier estimates. Univariate analyses were performed using Gray’s test for CIF, and the log-rank test for LFS and OS. Associations of patient and graft characteristics with outcomes were evaluated in multivariate analysis, using Cox proportional hazards model. All tests were two-sided. The type-1 error rate was fixed at 0.05 for determination of factors associated with time to event outcomes. Statistical analyses were performed with SPSS 22 (SPSS Inc./IBM, Armonk, NY) and R 3.2.2 (R Development Core Team, Vienna, Austria) software packages.

To allow for potential confounding factors between treatments that could influence outcome, propensity score matching was also performed, using the nearest neighbor matching and exact matching for patient age and cytogenetics group. The following factors were included in the propensity score model: patient age (less or more than 50 years), year of transplant, cytogenetics group, patient and donor CMV serology, female donor to male recipient vs other combination, time from diagnosis to transplant, Karnofsky performance status less or more than 90% at HSCT, and use of in vivo T cell depletion and conditioning (MAC/RIC/FLAMSA). Owing to the significant differences in baseline characteristics between the MSD and UD groups, caliper matching was fixed to 0.2. The purpose of the propensity score matching strategy was to reduce confounding effects of these variables and strengthen causal inferences. Propensity score analysis was performed using the “MatchIt” (Ref: Package “MatchIt”. 2015 (accessed: 18 May 2015). http://cran.project.org/web/packages/MatchIt/MatchIt.pdf). Comparisons between the two match-paired groups were stratified on matching group for taking into account for association using either mixed effects Cox model.

## Results

### Patients, disease and transplant characteristics

We obtained data from 211 reporting centers (Additional file 1), and 104 of whom used both types of donors. Patient and disease characteristics are summarized in Table 1. Six hundred sixty patients received a MSD and 381 patients an UD (296 with an HLA-matched 10/10, and 85 with a mismatched 9/10). The two patient cohorts were different for several variables (Table 1). The median year of transplant in the MSD group was 2007 (range 2000–2013), whereas patients with UD underwent HSCT more recently (median 2010, range 2000–2013; *p* < 10^−5^). Median follow-up for all patients was 39 months (32.5–47.5) but median follow-up was longer in the MSD group (48 months [range, 40.1–157.2]) than in the UD group (30 months [24.7–36.2], *p* = 0.04). All patients were refractory in active phase of disease at time of HSCT as per the study inclusion criteria. Median age was higher in the UD group (50.5 years (18–74) vs 47.7 years (18–74) in the MSD group, *p* = 0.006). The median time from diagnosis to HSCT was similar in the two groups (110 days [60–180] vs 111 days [60–178], respectively, *p* = 0.33). In the MSD group, 56.5% received a MAC regimen, 29.2% a RIC regimen, and 14.3% a sequential conditioning regimen mainly FLAMSA (fludarabine, cytarabine, and amsacrine) followed by conditioning regimen, while in the UD group, 44.4% received a MAC regimen, 24.4% a RIC regimen, and 31.2% a sequential conditioning regimen (*p* < 10^−4^). The MSD group contained more cytomegalovirus-positive recipients and recipients with cytomegalovirus-positive donors than did the UD group (Table 1). Cyclosporine and methotrexate were used as the main graft-versus-host disease prophylaxis in the MSD group. The proportion of patients who received in vivo T cell depletion significantly differed between the two groups (34.4% in the MSD group and 80.5% in the UD group, *p* < 10^−5^). PBSC was by far the main stem cell source in both groups (92% in the MSD group vs 94.8% in the UD group, *p* = 0.09) (Table 1).

**Table 1:** Baseline characteristics of patients

	MSD	UD	*p* value
Size, *n*	660	381	
Centers, *n*	199	104	
Median follow-up, months (range)	48 (40.1–157)	30 (27-36)	0.04
Year of Tx (range)	2007 (2000–13)	2010 (2000–13)	<10^−5^
Interval diagnosis to Tx, day (range)	110 (60–180)	111 (60–178)	0.33
Patient sex, *n* (%)			
Male	393 (59.5)	212 (55.8)	0.24
Female	267 (40.5)	168 (44.2)	
Donor sex, *n* (%)			
Male	347 (53)	253 (71)	<10^−5^
Female	308 (47)	103 (29)	
Cytogenetics, *n* (%)			0.02
Good	13 (4.9)	4 (2.9)	
intermediate	170 (64.2)	71 (51.8)	
Poor	82 (30.9)	62 (45.3)	
Unknown/failed	395	244	
KPS, *n* (%)			
<90%	183 (29.8%)	119 (33%)	0.30
≥90%	431 (70.2%)	242 (67%)	
Female D to male R, *n* (%)			
No	467 (71.3)	306 (86.2)	<10^−5^
Yes	188 (28.7)	49 (13.8)	
CMV patient, *n* (%)			
Negative	162 (30.5)	148 (40)	0.003
Positive	369 (69.5)	222 (60)	
CMV donor, *n* (%)			
Negative	203 (38.8)	204 (54.4)	<10^−5^
Positive	320 (61.2)	171 (45.6)	
Source of stem cell, *n* (%)			
BM	53 (8)	20	0.09
PBSC	607 (92)	361 (94.8)	
Conditioning regimen, *n* (%)			
MAC	373 (56.5)	169 (44.4)	<10^−4^
RIC	193 (29.2)	93 (24.4)	
Sequential strategy	94 (14.3)	119 (31.2)	
In vivo T depletion, *n* (%)			
No	357 (65.6)	73 (19.5)	<10^−5^
Yes	187 (34.4)	302 (80.5)	
GVHD prophylaxis, *n* (%)			
CsA alone	85 (12.9)	44 (11.5)	
CsA+ MTX	256 (38.8)	93 (24.5)	
CsA+ MMF + other	165 (25)	194 (50.9)	

*Abbreviations*: *BM* bone marrow, *CsA* cyclosporine, *D* donor, *KPS* Karnofsky Performance Status, *MAC* myeloablative conditioning regimen, *MSD* matched sibling donor, *MMF* mycophenolate mophetyl, *MTX* methotrexate, *PBSC* peripheral blood stem cell, *R* recipient, *RIC* reduced intensity conditioning regimen, *Tx* transplantation, *UD* unrelated donor, *GVHD* graft-versus-host disease

### Engraftment, GvHD, and response

Cumulative incidence (CI) of neutrophil engraftment was similar with a MSD and UD, 94.2 and 95.2%, respectively (*p* = 0.5). Day of absolute neutrophil count ≥500 cells per μL did not differ as well between HSCT from MSD vs UD, 15 days (6–43) and 15 days (1–43) days after HSCT, respectively (*p* = 0.76) (Table 2).

**Table 2:** Transplantation outcomes

	MSD (*n* = 660)	UD (*n* = 381)	*p* value
Engraftment	606 (94.2%)	359 (95.2%)	0.5
Acute GVHD			0.012
Grade 0–I	447 (72.1%)	236 (64.5%)	
Grade II–IV	173 (27.9%)	130 (35.5%)	
Outcome at 2 years			
Leukemia-free survival	25.3% (21.6–28.9)	28.3% (23.3–33.3)	0.56
Overall survival	30.9% (27–34.9)	34.3% (29–39.7)	0.57
Relapse	53.7% (49.6–57.7)	46.4% (40.9–51.7)	0.04
Non-relapse mortality	21% (17.9–24.4)	25.1% (21.7–28.6)	0.11
Chronic GVHD	28.9% (24.9–33)	25.8% (20.8–31)	0.77

Data are *n* (%) or *n* (%; 95% CI)

*MSD* matched sibling donors, *UD* unrelated donors, *GVHD* graft-versus-host disease

As expected, lower incidence of all grades aGVHD was observed post-HSCT with a MSD than a UD; indeed, CI of grades II–IV were 27.9 and 35.5%, respectively (*p* = 0.012). However, no difference was observed in the rates of severe grade III–IV aGVHD (12.3% in the MSD group and 16.1% in the UD group, (*p* = 0.09) (Table 2)).

At 2 years, the cumulative incidence of cGVHD was similar in the two groups (28.9 and 25.8%, respectively, *p* = 0.56) (Table 2). T cell in vivo depletion mostly represented by ATG was the only favorable factor to prevent cGVHD (HR = 0.51, 95% CI, 0.35–0.74, *p* = 0.001) (Table 3). When investigating the effect of cGVHD on the incidence of relapse using Cox with time-dependent variables (univariate analysis), we found a significant association between the two events (HR = 0.60, 95% CI, 0.45–0.81, *p* = 0.001).

**Table 3:** Multivariate analysis for LFS, OS, RI, NRM, and cGVHD

	HR	95% CI		*p* value
LFS				
UD vs MSD	0.96	0.79	1.17	0.68
Age ≥50 years	1.17	0.98	1.40	0.09
Year of Tx > median	1.02	0.85	1.23	0.83
Cytogenetics				
Poor vs intermediate	1.61	1.24	2.09	<10^−3^
Missing vs intermediate	1.18	0.96	1.46	0.11
CMV patient positive	1.19	0.98	1.44	0.09
CMV donor positive	0.99	0.82	1.19	0.90
Female to male	1.03	0.84	1.26	0.77
Diag to Tx > median	1.21	1.02	1.44	0.03
KPS > 90%	0.67	0.56	0.80	<10^−5^
In vivo T depletion	1.02	0.84	1.26	0.81
Conditioning regimen (ref = MAC)				
RIC vs MAC	0.89	0.73	1.08	0.25
Sequential strategy vs MAC	0.91	0.69	1.21	0.53
OS				
UD vs MSD	1.02	0.83	1.25	0.87
Age ≥50 years	1.33	1.10	1.61	0.004
Year of Tx > median	1.02	0.84	1.24	0.83
Cytogenetics				
Poor vs intermediate	1.55	1.19	2.03	0.001
Missing vs intermediate	1.23	0.99	1.53	0.06
CMV patient positive	1.28	1.04	1.58	0.02
CMV donor positive	0.97	0.80	1.19	0.79
Female to male	1.06	0.86	1.32	0.57
Diag to Tx > median	1.20	1.00	1.44	0.05
KPS >90%	0.65	0.54	0.79	<10^−5^
In vivo T depletion	0.94	0.76	1.16	0.55
Conditioning regimen (ref = MAC)				
RIC vs MAC	0.83	0.67	1.02	0.08
Sequential strategy vs MAC	0.81	0.60	1.09	1.16
RI				
UD vs MSD	0.89	0.71	1.12	0.33
Age ≥50 years	0.99	0.80	1.22	0.94
Year of Tx >median	0.95	0.77	1.18	0.66
Cytogenetics				
Poor vs intermediate	1.74	1.30	2.33	<10^−3^
Missing vs intermediate	1.10	0.86	1.40	0.45
CMV patient positive	1.07	0.86	1.33	0.56
CMV donor positive	0.97	0.78	1.20	0.75
Female to male	1.08	0.86	1.37	0.51
Diag to Tx > median	1.29	1.06	1.58	0.01
KPS >90%	0.77	0.62	0.95	0.01
In vivo T depletion	1.10	0.87	1.40	0.43
Conditioning regimen (ref = MAC)				
RIC vs MAC	1.03	0.81	1.29	0.83
Sequential strategy vs MAC	0.98	0.71	1.35	0.89
NRM				
UD vs MSD	1.30	0.91	1.86	0.14
Age ≥50 years	1.77	1.27	2.47	0.001
Year of Tx > median	1.18	0.84	1.64	0.33
Cytogenetics				
Poor vs intermediate	1.53	0.91	2.59	0.11
Missing vs intermediate	1.44	0.98	2.13	0.06
CMV patient positive	1.68	1.14	2.47	0.01
CMV donor positive	1.06	0.75	1.49	0.76
Female to male	1.01	0.70	1.47	0.95
Diag to Tx > median	1.06	0.77	1.45	0.72
KPS >90%	0.48	0.35	0.66	<10^−5^
In vivo T depletion	0.89	0.62	1.29	0.54
Conditioning regimen (ref = MAC)				
RIC vs MAC	0.59	0.41	0.85	0.005
Sequential strategy vs MAC	0.82	0.48	1.38	0.45
cGVHD				
UD vs MSD	1.22	0.86	1.74	0.26
Age ≥50 years	1.06	0.76	1.47	0.73
Year of Tx > median	1.06	0.76	1.48	0.72
Cytogenetics				
Poor vs intermediate	1.01	0.64	1.59	0.96
Missing vs intermediate	0.82	0.58	1.16	0.26
CMV patient positive	1.24	0.87	1.75	0.23
CMV donor positive	1.07	0.76	1.51	0.70
Female to male	1.53	1.07	2.19	0.02
Diag to Tx > median	0.85	0.62	1.17	0.32
KPS >90%	1.01	0.72	1.41	0.97
In vivo T depletion	0.51	0.35	0.74	0.001
Conditioning regimen (ref = MAC)				
RIC vs MAC	1.20	0.83	1.73	0.32
Sequential strategy vs MAC	1.07	0.63	1.80	0.81

*Abbreviations*: *CI* confidence interval, *Diag* diagnosis, *GVHD* graft-vs-host disease, *HR* hazard ratio, *KPS* Karnofsky Performance Status, *LFS* leukemia-free survival, *MAC* myeloablative conditioning, *MSD* matched sibling donor, *NRM* non-relapse mortality, *OS* overall survival, *RIC* reduced intensity conditioning, *Tx* transplantation, *UD* unrelated donor

In terms of response, after MSD and UD, 443 patients (71%) in the MSD group and 247 (69%) patients in the UD obtained a CR after HSCT, while 148 (24%) and 93 (26%) did not achieved complete remission. Thirty-three (5.3%) patients in the MSD group and 20 (5.6%) in the UD group were not evaluable for response because of early death, respectively (*p =* 0.73).

### LFS, OS, RI, and NRM

**Fig. 1:**
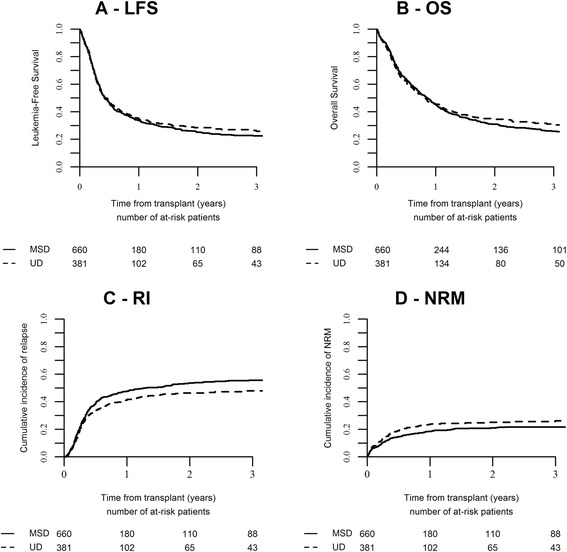
Probability of **a** leukemia-free survival (LFS), **b** overall survival (OS), **c** relapse incidence (RI), and **d** non-relapse mortality (NRM) in allografted patients with PRF-AML

In univariate analysis, LFS at 2 years was 25.3% in the MSD group vs 28.3% in the UD group, respectively (*p* = 0.56) (Fig. 1a). In univariate analysis, in intermediate cytogenetic group, LFS between the two types of donors MSD and UD was 29.5% (22.5–36.5) and 35.7% (24.2–47.2) (*p* = 0.34), respectively In the group with poor cytogenetic, LFS at 2 years was 16% (7.5–24.4) in patients receiving a MSD and 19.8% (9.4–30.1) in patients with an UD (*p* = 0.37); OS at 2 years was 21% (11.4–30.7) in the MSD group and 25.2% (13.9-36.5) in the UD group (*p* = 0.89). Multivariate analysis showed lower LFS in patients who had a poor cytogenetics compared to those with intermediary risk (HR = 1.61, 95% CI, 1.24–2.09, *p* = 0.0004) and when transplant was performed above the median of 110 days from diagnosis (HR = 1.21, 95% CI, 1.02–1.44, *p* = 0.03), whereas KPS at transplant ≥90% was associated with better LFS (HR = 0.67, 95% CI, 0.56–0.80, *p* = 0.0001). We noted no effect for donor types, year of transplant, patient or donor cytomegalovirus (CMV) status, in vivo T depletion, and type of conditioning regimen (Table 3).

OS at 2 years did not differ between the two groups (30.9% in the MSD group vs 34.3% in the UD group (*p* = 0.57)) (Fig. 1b). In univariate analysis, in the intermediate cytogenetic group, OS did not differ statistically between the two types of donors MSD and UD (37.1% (29.6–44.5) and 42.6% (30.7–54.4) respectively, *p* = 0.226). In the group with poor cytogenetic, OS at 2 years was 21% (11.4–30.7) in the MSD group and 25.2% (13.9–36.5) in the UD group (*p* = 0.89). OS at 2 years did not statistically differ between the group with in vivo T cell-depleted graft and no T cell-depleted graft (*p* = 0.49) (Additional file 1: Table S1). Looking at the conditioning regimen, no difference was observed between MAC, RIC, and sequential strategy (*p* = 0.602) (Additional file 1: Table S1). These results were confirmed by multivariate analysis. In multivariate analysis, four predictive factors were associated with lower OS: age >50 years, cytogenetics (poor vs intermediate), time from diagnosis to transplant, and CMV-positive status, whereas KPS at transplant ≥90% was associated with a better OS (Table 3).

We noted a higher RI in the MSD group compared to the UD group (53.7 and 46.4%, *p* = 0.04; respectively) (Table 2, Fig. 1c). However, in multivariate analysis for RI, only cytogenetics (poor vs intermediate) and time from diagnosis to transplant were the only risk factors associated with increased RI [(HR = 1.74, 95% CI, 1.30–2.33, *p* = 0.0002) (HR = 1.29, 95% CI, 1.06–1.58, *p* = 0.01), respectively] whereas KPS at transplant was a protective factor (HR = 0.77, 95% CI, 0.62–0.95, *p* = 0.01) (Table 3).

No difference in NRM was noted between the two groups in univariate analysis (Table 2, Fig. 1d). However, multivariate analysis demonstrated that patient age (≥50 years), and CMV-positive status were associated with higher NRM (HR = 1.77, 95% CI, 1.27–2.47, *p* = 0.001; HR = 1.68, 95% CI, 1.14–2.47, *p* = 0.008), while RIC regimen compared to MAC regimen was the only factor associated with lower NRM (HR = 0.59, 95% CI, 0.41–0.85, *p* = 0.005) (Table 3). We noted no effect for donor types.

Finally, in order to reduce the effects of confounding factors, we did a propensity score analysis. Using a caliper of 0.2, we were able to match 221 MSD and 221 UD on the following variables: patient age (less or more than 50 years), year of transplant, cytogenetics group, patient and donor CMV serology, female donor to male recipient versus other combination, time from diagnosis to transplant, Karnofsky performance status less or more than 90% at HSCT, and use of in vivo T cell depletion and conditioning (MAC/RIC/FLAMSA). The results of the Cox analysis were confirmed in this subpopulation of patients with balanced characteristics as described in Table 4.

**Table 4:** Propensity score analysis for LFS, OS, RI, NRM and cGVHD

	MSD	UD	*p* value (frailty)
LFS	25.7% (19.1–32.3)	30.1% (23.4–36.8)	0.56
OS	31.3% (24.1–38.6)	38.2% (31.1–45.4)	0.94
RI	54.3% (46.8–61.1)	45.6% (38.3–52.5)	0.11
NRM	20% (14.6–26.1)	24.1% (18.4–30.2)	0.19
cGVHD	24.2% (17.8–31.2)	30.3% (23.4–37.5)	0.08

*Abbreviations*: *cGVHD* chronic graft-vs-host disease, *LFS* leukemia-free survival, *MSD* matched sibling donor, *NRM* non-relapse mortality, *OS* overall survival, *UD* unrelated donor

### Causes of death

AML was the most common cause of death (64.9% in the MSD group and 55.3% in the USD group). GVHD represented 14.2% of death causes in the MSD group and 16.1% in the UD group. Infection was the death cause in 10.8 and 19.4% for the MSD and UD groups, respectively.

## Discussion

In the present study, we addressed the topic of HSCT for patients with PRF-AML a devastating medical condition with no other therapeutic option for cure. Specifically, we compared the transplantation outcomes after MSD versus UD (10/10 or 9/10). Our cohort included 1041 patients: 660 received MSD HSCT and 381 a UD HSCT (296 10/10 matched UD HSCT, and 85 9/10 matched UD). We were able to show about 25% LFS and 30% OS post-HSCT for this high-risk advanced disease with no difference between sibling vs unrelated HSCT. Despite higher rates of grade II–IV aGVHD in the UD group, neither the 2-year cumulative incidence of severe grade III–IV aGVHD, nor cGVHD or the 2-year NRM rates differed significantly between the two groups (Tables 2 and 3). Although we could hypothesize a stronger graft-versus-leukemia effect post UD compared to MSD, we did not observed difference in terms of RI or LFS. Even in the setting of maximum HLA disparity namely haploidentical transplants, we could not observed lower relapse rate as compared to HLA-matched sibling HSCT [20, 31, 32]. One theoretic explanation is that due to the very aggressive biology of the leukemia being refractory to chemotherapy disease, progression and relapse occur early after transplant as even the MAC or FLAMSA conditioning are unable to control the disease while (i) it takes time (up to 9 months) for the GVL effect to be established and to work against the leukemia and (ii) in order to be effective, a state of minimal residual disease or at least substantial reduction in the leukemic tumoral mass need to be achieved [33, 34].

In our study, 27.5% of patients received a RIC regimen. As it is PRF, NRM was significantly lower in the RIC group compared to the MAC group, but there were no statistically differences in LFS, OS, and RI. This study had included 213 HSCT patients who received sequential approach with aplasia-inducing chemotherapy followed by conditioning regimen. Schmid et al. found a 2-year OS of 40% and a 2-year NRM of 22% using FLAMSA followed by TBI/cyclophosphamide in patients with refractory AML who received a MSD or a UD [35]. Stelljes et al. had shown previously that the 2-year OS with RIC in AML patients in complete remission versus untreated or refractory disease from related or unrelated donors is 81 and 21%, respectively [32, 36]. This underlines the potential effect of prior leukemic burden reduction to improve survival. In the current study, no outcome differences were found between MAC, RIC, or sequential regimens. Due to the aggressive biology of the leukemia and the possible refractoriness to chemotherapy of the malignant leukemic clone, it is conceivable that even the most intense chemotherapy conditioning is unable to induce remission or even transient response which allow sufficient time for the alloreactive cells to mediate the GVL effect [34]. Future prospective studies should more specifically address the outcome differences between these approaches.

Our data identified that proceeding to HSCT as soon as possible was one of the most important factors determining the PRF-AML outcome. Indeed, time from diagnosis to transplant longer than the median (110 days) was a negative prognostic factor for LFS, OS, and RI in multivariate analysis. These data are consistent with the study of Craddock et al. [12] and emphasize the urgent need of searching for an UD for PRF-AML patients who lack a matched sibling donor. We may add that this is a factor we can influence as oppose to most of other factors like disease biology, cytogenetics, age, and comorbidities. Thus, we recommend in patients that failed two lines of therapy not to try additional lines to achieve CR but to do a fast search and to proceed to transplant.

Cytogenetic features of the AML represent a major prognostic factor in LFS, RI, and OS [37, 38]. Duval et al. reported that poor-risk cytogenetics was an adverse pre-HSCT variable in patients with PRF-AML or relapsed AML and who underwent a MAC regimen [6]. Thereby, PRF-AML with poor cytogenetic characteristics were associated with statistically significant lower LFS, higher RI, and lower OS at 2 years in multivariate analysis. These data pave the road for investigating more intensive additional approaches relying on sequential conditioning regimens and/or post-transplant treatments such as 5-azacytidine, prophylactic donor lymphocytes infusions, or targeted therapy in order to further improve in the prognosis of this devastating subgroup of patients [39].

Several studies have investigated the prognostic impact of KPS at transplant [6, 40, 41]. KPS was independently correlated with toxicities, NRM, and overall mortality [40]. In our study, KPS at HSCT was of prognostic value. Indeed, KPS, when stratified into scores of less than 90% and greater than 90% at HSCT, had a significant predictive value for LFS, OS, RI, and NRM. This simple parameter is a powerful predictor of post-HSCT outcomes and may therefore contribute to guide our practical management of PRF-AML.

Being a retrospective study has few limitations: patient characteristics vary among the groups for several factors including year of transplant, age, patient and recipient CMV status, and cytogenetic risk. There is a relative inherent selection process for HSCT in our study, since the patients corresponded to a subgroup who fitted the criteria to undergo HSCT. Also, we do not have information about why patients were allocated to a specific donor in the registry and distinguishing the choice of the donor from the role of a potential center effect is difficult. Finally, number of circulating and bone marrow blasts at time of HSCT are missing in substantial number of patients. However, the aim of this analysis was to compare the two types of donors using EBMT registry data. The design of the study and inclusion criteria were intended to answer this clinical question and therefore are not adapted to develop a prognostic score based on information that is not routinely collected in the registry. Unfortunately, no ongoing trials are comparing outcomes after MSD or UD for PRF-AML. Therefore, in the absence of any prospect of such comparative studies, our data suggest that both donors are equally effective in these very high risk patients.

## Conclusions

Our results suggest that, when an HLA-identical sibling donor is not available for an adult with PRF-AML who is otherwise a candidate for HSCT, a 10/10 or 9/10 UD may be used with the expectation of similar rates of NRM, LFS, and OS at 2 years. However, despite this expanded use, as many as one-third of patients will not get a UD donor source, so that alternative options such as haploidentical donors and use of cord blood stem cells may need consideration and evaluation. In these circumstances, we would recommend to rather proceed fast to transplant than multiplying the number of lines of chemotherapy that would increase toxicity without achieving an anti-leukemic response and thus will hamper the chance to reach HSCT that may rescue about 25% of the patients. Disease relapse remains the most common cause of treatment failure after HSCT for this group of patients with PRF-AML. Potential approaches to reduce this high risk of AML relapse following HSCT could be prophylactic or preemptive therapy. Few prospective single-arm studies investigating hypomethylating agents (5-azacytidine or decitabine) as consolidation therapy for patients with AML or myelodysplastic syndrome after HSCT have been published [42–46]. Given the limited number of patients and the lack of a control arm, a definitive ranking of outcome results is of course difficult so far but they suggested a potential benefit impact. Panobinostat, a potent inhibitor of deacetylases, maintenance post-HSCT was reported to be feasible and associated with a low relapse rate [47]. Sorafenib maintenance has been recently reported in 27 patients with FTL3-ITD+ with encouraging results [48]. In all, these data suggest that primary refractory AML patients should be transplanted as quickly as possible with either a MSD or an UD and maintenance therapy should be a very promising approach for these very high risk patients.

## Additional file

Additional file 1 Table 1S: Conditioning regimens: univariate analysis for RI, NRM, LFS, OS, cGVHD. (DOCX 20 kb)

## Abbreviations

aGVHB: Acute graft-versus-host disease
ALWP: Acute leukemia working party
AML: Acute myeloid leukemia
BM: Bone marrow
BU: Busulfan
cGVHD: Chronic graft-versus-host disease
CIF: Cumulative incidence functions
CMV: Cytomegalovirus
CR: Complete remission
EBMT: European Society of Blood and Marrow Transplantation
FLAMSA: Fludarabine, cytarabine, and amsacrine
GVL: Graft-versus-leukemia effect
HLA: Human leukocyte antigen
HSCT: Allogeneic stem cell transplantation
KPS: Karnofsky performance status
LFS: Leukemia-free survival
MAC: Myeloablative conditioning regimen
MSD: Matched sibling donor
NRM: Non-relapse mortality
OS: Overall survival
PBSC: Peripheral blood stem cell
PRF-AML: Primary refractory acute myeloid leukemia
RI: Relapse incidence
RIC: Reduced intensity conditioning regimen
UD: Unrelated donor
