# Supplementary material for: Comparison of matched sibling donors versus unrelated donors in allogeneic stem cell transplantation for primary refractory acute myeloid leukemia: a study on behalf of the Acute Leukemia Working Party of the EBMT
Source: J Hematol Oncol. 2017 Jun 24;10:130. doi: 10.1186/s13045-017-0498-8 (PMC5483262; doi:10.1186/s13045-017-0498-8)
Supplement: Additional file 1 Table 1S: — Conditioning regimens: univariate analysis for RI, NRM, LFS, OS, cGVHD. (DOCX 20 kb) [file 13045_2017_498_MOESM1_ESM.docx]

**Additional file 1**

**Table 1S: Conditioning regimens: univariate analysis for RI, NRM, LFS, OS, cGVHD**

| 2 years results | RI | NRM | LFS | OS | cGVHD |
| --- | --- | --- | --- | --- | --- |
| RIC | 53.6% (47.7-59.2) | 18.8% (10.9-28.3) | 27.6% (22.4-32.8) | 33.5% (28-39.1) | 27.5% (22.3-33) |
| MAC | 49.1% (44.7-53.3) | 24.8% (15.8-34.8) | 26% (22.1-30) | 30.3% (26.1-34.5) | 29.5% (25.1-34) |
| Sequential strategy | 53.1% (43.3-62) | 22.3% (13.8-32.2) | 24.5% (16.3-32.8) | 36.9% (27.5-46.2) | 21.1% (13.4-29.8) |
| p | 0.783 | 0.516 | 0.276 | 0.602 | 0.116 |
| Abbreviations: cGVHD= chronic graft-vs-host disease. LFS=leukemia-free survival. MAC=myeloablative conditioning. MSD=matched sibling donor. NRM=non-relapse mortality. OS=overall survival. RIC=reduced intensity | | | | | |
|  |  |  |  |  |  |
|  |  |  |  |  |  |
|  |  |  |  |  |  |

" Comparison of matched-sibling donors versus unrelated donors in allogeneic stem cell transplantation for primary refractory acute myeloid leukemia: A study from the Acute Leukemia Working Party of the EBMT " by Eolia Brissot,

List of participating centers, for acknowledgement

University of Muenster, Dept. of Hematol./Oncol., Muenster, Germany; Universitaetsklinikum Dresden, Medizinische Klinik und Poliklinik I, Dresden, Germany; Deutsche Klinik fuer Diagnostik, KMT Zentrum, Wiesbaden, Germany; University of Freiburg, Dept. of Medicine -Hematology, Oncology, Freiburg, Germany; Klinikum Grosshadern, Med. Klinik III, Munich, Germany; Hannover Medical School, Department of Haematology, Hemostasis, Oncology, and Stem Cell Transplantation, Hannover, Germany; Klinikum Nuernberg, 5. Medizinische Klinik, BMT-Unit, Nuernberg, Germany; University Hospital Eppendorf, Bone Marrow Transplantation Centre, Hamburg, Germany; Klinik fuer Innere Medzin III, Universitätsklinikum Ulm, Ulm, Germany; Universitaetsklinikum Wuerzburg, Med. Klinik und Poliklinik II, Wuerzburg, Germany; Chaim Sheba Medical Center, Chaim Sheba Medical Center, Dept. of Bone Marrow Transplantation, Tel-Hashomer, Israel; Universitaet Tuebingen, Medizinische Klinik, Abteilung II, Tuebingen, Germany; CHU Bordeaux, Hôpital Haut-leveque, Pessac, France; Medizinische Universitaet Wien, Klinik fuer Innere Medizin I, Knochenmarktransplantation, Vienna, Austria; Charité - Campus Benjamin Franklin, Universitaetsmedizin Berlin, Medizinische Klinik III - Hämatologie u Onkologie, Berlin, Germany; Asklepios Klinik St. Georg, Department of Haematology, Hamburg, Germany; University Medical Center Schleswig-Holstein, Campus Kiel, División of Stem Cell Transplantation and Immunotherapy, Kiel, Germany; Institute of Hematology and Blood Transfusion, Servicio de Hematología, Prague, Czech Republic; University Regensburg, Dept. of Hematology and Oncology, Regensburg, Germany; Klinikum Augsburg, II Medizinische Klinik, Augsburg, Germany; King Faisal Specialist Hospital & Research Centre, Oncology (Section of Adult Haematolgy/BMT), Riyadh, Saudi Arabia; University of Heidelberg, Medizinische Klinik u. Poliklinik V, Heidelberg , Gernamy; University Medical Center Mainz, Department of Hematology, Oncology and Pneumology, Germany, Germany; Charité Universitaetsmedizin Berlin, Campus Virchow Klinikum, Medizinische Klinik m. S. Hämatologie/Onkologie, A, Berlin, Germany; Hospital Clinic, Institute of Hematology & Oncology, Dept. of Hematology, Barcelona, Spain; Programme de Transplantation&Therapie Cellulaire, Centre de Recherche en Cancérologie de Marseille, Institut Paoli Calmettes, Marseille, France; University Hospital Leipzig, Division of Haematology & Oncology, Leipzig, Germany; Elisabethinen-Hospital, I. Internal Department, Linz, Austria; Philipps Universitaet Marburg, University Hospital Giessen and Marburg, Campus Marburg, Marburg, Germany; George Papanicolaou General Hospital, Haematology Department / BMT Unit, Thessaloniki, Greece; Nottingham University, Hucknall Road, Nottingham, United Kingdom; St. Bartholomew`s and The Royal London NHS Trust, London, United Kingdom; University Hospital Erlangen, Dept. of Internal Medicine 5, Erlangen, Germany; University Hospital Gasthuisberg, Dept. of Hematology, Leuven, Belgium; Rambam Medical Center, Dept. of Hematology & BMT, Haifa, Israel; University Hospital Birmingham NHSTrust, Queen Elizabeth Medical Centre, Edgbaston, Birmingham, United Kingdom; Heinrich Heine Universitaet, Klinik für Hämat,Onkol,Klin.Immun., Duesseldorf, Germany; Azienda Ospedaliera Papa Giovanni XXIII, Hematology and Bone Marrow Transplant Unit, Bergamo, Italy; University Hospital La Fe, Hematology Department, Valencia, Spain; , Nouvel Hopital Civil, Strasbourg, France; Fiona Stanley Hospital, Hematology Department, Perth, Australia; Klinikum Oldenburg, Abt. Onkologie/Hämatologie, Oldenburg, Germany; Tel Aviv Sourasky Medical Center, Blood and Bone Marrow Transplantation, Tel-Aviv, Israel; University Hospital, Hematology, Basel, Switzerland; University Hospital, Clinic of Hematology, Zürich, Switzerland; Hadassah University Hospital, Dept. of Bone Marrow Transplantation, Jerusalem, Israel; BMT unit, Clinica Ematologica, Fondazione IRCCS Policlinico San Matteo, Pavia, Italy; Medical University Graz, LKH - University Hospital Graz, Division of Haematology, Graz, Austria; Beilinson Hospital, Hematology and BMT Department, Petach-Tikva, Israel; Klinikum Rechts der Isar, III Med Klinik der TU, Munich, Germany; University Med. Center, Department of Hematology, Ljubljana, Slovenia; Hôpital Percy, Hematology Department, Clamart, France; Centre Hospitalier Lyon Sud, Pavillon Marcel Bérard -Bat 1G, Service Hematologie, Lyon, France; Southampton General Hospital, Haematology, Oncology, & Paediatrics, Dept. of Haematology, Southampton, United Kingdom; GKT School of Medicine, Dept. of Haematological Medicine, London, United Kingdom; Hopital Saint Antoine, Department of Hematology, Paris, France; Turku University Hospital, TD7 (Stem Cell Transplant Unit), Turku, Finland; Western General Hospital, Dept. of Haematology, Edinburgh, United Kingdom; CHU Nantes, Dept. D`Hematologie, Nantes, France; Hospital Santa Creu i Sant Pau, Hematology Department, Barcelona, Spain; A.Z. Sint-Jan, Dept. of Hematology, Brugge, Belgium; Hospital de Gran Canaria `Dr Negrin`, Servicio de Hematología y Hemoterapia, Las_Palmas, Spain; Ospedale San Gerardo, Clinica Ematologica dell`Universita Milano-Biocca, Monza, Italy; Institut Universitaire du Cancer Toulouse, Oncopole, I.U.C.T-O, Toulouse, France; Ege University Medical School, Dept. of Hematology, Izmir, Turkey; Charles University Hospital, Dept. of Hematology/Oncology, Pilsen, Czech Republic; Central Clinical Hospital, The Medical University of Warsaw, Department of Hematology & Oncol, Warsaw, Poland; Robert_Bosch_Krankenhaus, Abt. Hämatologie / Onkologie, Stuttgart, Germany; Bone Marrow Transplant Unit L 4043, National University Hospital, Rigshospitalet, Copenhagen, Denmark; Cliniques Universitaires St. Luc, Dept. of Haematology, Brussels, Belgium; Bologna University, S.Orsola-Malpighi Hospital, Institute of Hematology & Medical, Oncology L & A Seràgnoli, Bologna, Italy; Hopital Henri Mondor, Sve d` Hematologie, Creteil, France; Hôpitaux Universitaires de Genève, Département des Spécialités de Médecine, Service d’Hématologie, Geneva , Switzerland; Skanes University Hospital, Dept. of Hematology, Lund, Sweden; Sahlgrenska University Hospital, Center for Hematopoietic Cell Transplantation, Hematology Section, Goeteborg, Sweden; CHU Nice - Hôpital de l`ARCHET I, Hematologie Clinique, Nice, France; IRCCS, Casa Sollievo della Sofferenza, Unit of Hematology and Bone Marrow Transplantation, San_Giovanni_Rotondo, Italy; Universitaetsklinikum Goettingen, Abteilung Hämatologie und Onkologie, Goettingen, Germany; University Hospital Maastricht, Dept. Internal Med.Hematology /Oncology, Maastricht, The Netherlands; Az. Ospedaliera S. Croce e Carle, Division of Hematology, Cuneo, Italy; U.O. Ematologia con Trapianto, Azienda Ospedaliero Universitaria Policlinico Bari, Bari, Italy; Umea University Hospital, Hematology, Umeå, Sweden; ICO – Hospital Duran i Reynals, L`Hospitalet de Llobregat, Barcelona, Spain; University of Saarland, University Hospital, Dept. of Internal Med., BMT Unit, Homburg, Germany; CHU Lapeyronie, Département d`Hématologie Clinique, Montpellier, France; Hôpital Necker, Service Hematologie Adulte, Paris , France; Centre National de Greffe de Moelle, Tunis, Tunisia; Institut Jules Bordet, Experimental Hematology, Brussels, Belgium; Hopital Jean Minjoz, Service d`Hématologie, Besancon, France; Department of Hematology, Clinic for Cancer, Surgery and Transplantation, Oslo University Hospital, Rikshospitalet, Oslo, Norway; University Hospital, Dept. of Bone Marrow Transplantation, Essen, Germany; Hopital La Miletrie, Head of the Bone Marrow TransplantUnit, Clinical Hematology, Poitiers, France; University Hospital, Dept. of Medicine, Uppsala, Sweden; University Hospital Innsbruck, Internal Medicine V (Hematology & Oncology), Innsbruck, Austria; Inst. Português de Oncologia do Porto, BMT Unit, Porto , Protugal; U.O.S.A Centro Trapianti e Terapia Cellulare, Azienda Ospedaliera Universitaria Senese, Policlinico S.Maria alle Scotte, Portugal, Italy; Univ. Est. de Campinas/TMO/UNICAMP, Cidade Universitaria `Zeferino Vaz`, Campinas, Brazil; Universitaetsklinikum Jena, Klinik für Innere Medizin II, (Abt. Hämatologie und Onkologie), Jena, Germany; Klinik fuer Knochenmarktransplantation, und Hämatologie/Onkologie GmbH, Idar-Oberstein, Germany; Hospital del SAS, Dept. of Hematology, Cádiz, Spain; ICO-Hospital Universitari Germans Trias i Pujol, Cattedra e Servizio di Ematologia, Barcelona, Spain; Ankara University Faculty of Medicine, Dept. of Hematology, Adult Stem Cell Transplantation Unit, Ankara, Turkey; CHRU, Service des Maladies du Sang, Angers, France; Ospedale La Maddalena - Dpt. Oncologico, Unità Operativa di Oncoematologia e, Trapianto di Midollo, Palermo, Italy; Azienda Ospedaliero Universitaria di Udine, Division of Hematology, Udine, Italy; Hospital Clínico, Servicio de Hematología, Salamanca, Spain; Hospital Universitario La Paz, Hematologia-Oncologia, Madrid, Spain; Hospital Morales Meseguer, Unidad de Trasplante de Médula Osea, Serv de Hemat, Murcia, Spain; University Hospital, Dept. of Hematology, Linköping, Sweden; Christie NHS Trust Hospital, Adult Leukaemia and Bone Marrow Transplant Unit, Manchester, United Kingdom; Hospital Gregorio Marañón, Sección de Trasplante de Medula Osea, Madrid, Spain; Antwerp University Hospital (UZA), Dept. of Hematology, Antwerp_Edegem, Belgium; Klinikum Chemnitz gGmbH, Klinik für Innere Medizin III, Chemnitz, Germany; Mazzoni Hospital, Haematology Service, Ascoli_Piceno, Italy; Clinical Hematology and BMT Unit, Targu-Mures, Romania; Leiden University Hospital, BMT Centre Leiden, Leiden, The Netherlands; Hopital St. Louis, Dept.of Hematology - BMT, Paris, France; Royal Free Hospital and School of Medicine, Department of Hematology, London, United Kingdom; Bone Marrow Transplant Unit, Beatson, West of Scotland Cancer Centre, Gartnaval General Hospital, Glasgow, United Kingdom; Academisch Ziekenhuis bij de Universiteit, van Amsterdam, Emma Kinderziekenhuis, Amsterdam, The Netherlands; Department of Haematology, Cancer and Haematology Centre, Churchill Hospital, Oxford, United Kingdom; Hope Directorate, St. James`s Hospital, Dublin, Ireland; Universite Paris IV, Hopital la Pitié-Salpêtrière, Hematologie Clinique, Paris, France; Hopital Bretonneau, Service d`Oncologie Médicale, Tours, France; CHU ESTAING, Service d’hématologie clinique Adulte et pédiatrie, Clermont-Ferrand, France; Hôpital HURIEZ, UAM allo-CSH, CHRU, Lille, France; Hospital Clínico Universitario, Servicio de Hematología, Valencia, Spain; Ospedale S. Camillo-Forlanini, Dept. of Hematology and BMT, Rome, Italy; Klinikum Karlsruhe gGmbH, III. Med. Klinik, Haematologie, Onkologie, Karlsruhe, Germany; Goethe-Universitaet, Medizinische Klinik II, Hämatologie, Medizinische Onkologie, Frankfurt am Main, Germany; Department of Haematology, University Hospital of Wales, Cardiff, United Kingdom; Azienda Ospedaliera Universitaria Careggi, Cell Therapy and Transfusion Medicine Unit, Firenze, Italy; Universita Cattolica S. Cuore, Istituto di Ematologia, Ematologia, Rome, Italy; Martin-Luther-Universitaet Halle-Wittenberg, Klinik für Innere Medizin IV, Halle, Germany; GATA BMT Center, Gülhane Military Medical Academy, Ankara, Turkey; U.O.D Trapianti di midollo osseo, A.O.R Villa Sofia-Cervello, Palermo, Italy; HUCH Comprehensive Cancer Center, Stem Cell Transplantation Unit, Helsinki , Finland; Pesaro Hospital, Hematology & Transplant Centre, Pesaro, Italy; University Hospital, Department of Haemato-Oncology, Olomouc, Czech Republic; Azienda Ospedaliera, Centro Unico Regionale Trapianti, Reggio_Calabria, Italy; VU University Medical Center, Department of Hematology (Br 250), Amsterdam, The Netherlands; University Hospital Brno, Dept. of Internal Med. - Hematooncology, Brno, Czech Republic; University Hospital, Department of Hematology and Transfusiology, Bratislava, Slovakia; Erciyes Medical School, Dept. of Hematology - Oncology, Kapadokya (Cappadocia) BMT Center, Kayseri, Turkey; Vilnius University Hospital Santariskiu Klinikos, Haematology, Oncology & Transfusion Center, Vilnius, Lithuania; Hospital Universitari Son Espases, Hematology Service, Palma_De_Mallorca, Spain; Charles University Hospital, 4th Department of Internal Medicine - Hematology, Hradec_Králové, Czech Republic; University of Napoli, `Federico II` Medical School, Division of Hematology, Napoli, Italy; Ospedale A. Businco Cagliari, Haematology & Transplant Centre Wilma Deplano, Cagliari, Italy; Azienda Policlinico Vittorio Emanuele, Programma di Trapianto Emopoietico Misto e Metropolitano Di Catania, Ospedale Ferrarotto, Catania, Italy; Ospedale San Raffaele s.r.l., Haematology and BMT, Milano, Italy; Plymouth Hospitals NHS Trust, Derriford Hospital, Plymouth, United Kingdom; H SS. Antonio e Biagio, Haematology Department, Alessandria, Italy; Medical Park Hospitals, Stem Cell Transplant Unit, Antalya, Turkey; First Affiliated Hospital of Soochow University, Department of Hematology, Suzhou, China; Sana Klinikum Hameln-Pyrmont, Abt. Haem/Onkologie, Hameln, Germany; USD Trapianti di Midollo, Adulti, Universita di Brescia, Brescia, Italy; Adnan Menderes University Med. Faculty, Hematology Department, Aydin, Turkey; Haukeland University Hospital, Department of Haematology, Bergen, Norway; Ospedale San Martino, Department of Haematology II, Genova, Italy; University College London Hospital, Department of Haematology, London, United Kingdom; S.S.C.V.D Trapianto di Cellule Staminali, A.O.U Citta della Salute e della Scienza di Torino, Presidio Molinette, Torino, Italy; Hospital de la Princesa, Department of Hematology, Madrid, Spain; University Medical Centre, Dept. of Haematology, Utrecht, The Netherlands; Hospital U. Marqués de Valdecilla, Servicio de Hematología-Hemoterapia, Santander, Spain; Ospedale Civile, Dipartimento di Ematologia, Medicina Trasfusionale e Biotecnologie, Pescara, Italy; CHRU St. Etienne, Hopital Nord, Service d`Hematologie Clinique, Saint_Etienne, France; CHU CAEN, Institut d’hématologie de Basse-Normandie, Caen, France; Yorkshire Blood & Marrow Transplant Programme, Haematology Department, Level 3, Bexley Wing, St James`s Institute of Oncology, Leeds, United Kingdom; Hopital A. Michallon, Department of Hematology, Grenoble, France; Adult HSCT unit, Northern Centre for Bone Marrow Transplantation, Freeman Hospital, Newcastle-Upon-Tyne, United Kingdom; Birmingham Heartlands Hospital, Department of Haematology, Birmingham, United Kingdom; Hospital San Maurizio, Dept. of Hematology - BMT Unit, Bolzano, Italy; Istituto Clinico Humanitas, Transplantation Unit, Department of Oncology and Haematology, Milano, Italy; Department of Internal Medicine, American University of Beirut Medical Center, Beirut, Lebanon; Bristol Royal Hospital for Children, Dept. of Paediatric Oncology/BMT, Bristol, United Kingdom; Ospedale Dell`Angelo, Hematology Department, Venezia, Italy; University of Cologne, I. Dept. of Medicine, Cologne, Germany; Azienda Ospedaliero Universitaria di Modena Policlinico, Ematologia, Modena, Italy; Dept. Haematology and Stem Cell Transplant, St. István and St. László Hospital, Budapest, Hungary; Hospital Univ. Virgen de las Nieves, Servicio de Hematología, Granada, Spain; Hospital Clinico Universitario, Servicio de Hematología, Santiago_De_Compostela, Spain; Clinic of Hematology, Military Medical Academy, Belgrade, Serbia and Montenegro; National Haematology Centre, Clinic Linezers, Riga, Latvia; Baskent University Hospital, Haematology Division, BMT Unit, Haemaology Reserach Laboratory, Training & Medical, Adana, Turkey; Manchester Royal Infirmary, Clinica Haematology Department, Manchester, United Kingdom; Klinikum Bremen-Mitte, Hämatologie / Onkologie, Klinik für Innere Medizin, Bremen, Germany; Hospital Ramón y Cajal, Servicio de Hematología, Madrid, Spain; Evangelismos Hospital, Division of Hematology, BMT Unit, Athens, Greece; Policlinico G.B. Rossi, Divisione di Ematologia, Unità di TMO, Verona, Italy; Shariati Hospital, Hematology-Oncology and BMT Research, Teheran, Iran; Hospital Universitario Central de Asturias, Oviedo, Spain; C.H.R.U de Brest, Service Onco-Hematologie, Brest, France; Hopital d`Enfants, Hematology, Vandoeuvre_Les_Nancy, France; Silesian Medical Academy, Univ. Dept. of Haematology and BMT, Katowice, Poland; University Hospital SPSK 1, Department of Hematology, Wroclaw, Poland; University of Liege, Dept. of Hematology, CHU Sart-Tilman, Liege, Belgium; Clinica Puerta de Hierro, Servicio de Hematologia y Hemoterapia, Madrid, Spain; Poznan University of Medical Sciences, Department of Hematology, Poznan, Poland; Fundación Favaloro, Buenos_Aires, Argentina; Ghent University Hospital, Haematology, Gent, Belgium; Haematology Department, St.Savvas Oncology Hospital, Athens, Greece; Schneider Children`s Medical Center of Israel, BMT Unit, Petach-Tikva, Israel; Ýstanbul Tip Fakultesi, Iç Hastaliklari ABD, Kemik iliði nakil unitesi, CAPA, Istanbul, Turkey; Sezione di Ematologia, Dipartimento di Medicina Clinica e Sperimentale, Università di Perugia, Ospedale Santa Maria della, Perugia, Italy; Military Institute of Health Services BMT Unit, Bone Marrow Transplantation Unit, Warsaw, Poland; Federal Centre of Heart, Blood and Endocrinology, named after V.A. Almazov, Dept. of Hematology, St._Petersburg, Russia; National Research Center for Hematology, Bone Marrow Transplantation, Moscow, Russia; Centre Henri Becquerel, Hematology, Rouen, France; University of Amiens: CHU Amiens, Service d`Hematologie, Amiens, France; St. Franziskus Hospital, Medizinische Klinik I, Flensburg, Germany;
